# Creativity and personality in classical, jazz and folk musicians

**DOI:** 10.1016/j.paid.2014.01.064

**Published:** 2014-06

**Authors:** Mathias Benedek, Barbara Borovnjak, Aljoscha C. Neubauer, Silke Kruse-Weber

**Affiliations:** a Department of Psychology, University of Graz, Austria; b Department of Instrumental- and Vocal Pedagogy, University of Music and Performing Arts Graz, Austria

**Keywords:** Music genre, Creativity, Personality, Divergent thinking, Music learning

## Abstract

The music genre of jazz is commonly associated with creativity. However, this association has hardly been formally tested. Therefore, this study aimed at examining whether jazz musicians actually differ in creativity and personality from musicians of other music genres. We compared students of classical music, jazz music, and folk music with respect to their musical activities, psychometric creativity and different aspects of personality. In line with expectations, jazz musicians are more frequently engaged in extracurricular musical activities, and also complete a higher number of creative musical achievements. Additionally, jazz musicians show higher ideational creativity as measured by divergent thinking tasks, and tend to be more open to new experiences than classical musicians. This study provides first empirical evidence that jazz musicians show particularly high creativity with respect to domain-specific musical accomplishments but also in terms of domain-general indicators of divergent thinking ability that may be relevant for musical improvisation. The findings are further discussed with respect to differences in formal and informal learning approaches between music genres.

## Highlights

• Creativity and personality of classical, jazz, and folk musicians was compared.
• Jazz musicians show higher divergent thinking ability.
• Jazz musicians accomplish more creative musical activities and achievements.
• Classical musicians show a high amount of practice and win more competitions.
• Folk musicians are more extraverted and publish more musical productions.

## 1 Introduction

Within the field of music, jazz is commonly considered as a particularly creative discipline (e.g., Barrett, 1998). This appraisal is related to the fact that jazz music involves a high degree of improvisational playing. Jazz improvisation can range from the simple embellishment of the melody of the theme to e.g. the continuous extemporization of entirely new melodies that fit to the sequence of chords (Johnson-Laird, 2002; Pressing, 1988). Jazz musicians who are highly skilled in improvising hence may possess traits that are different from those of musicians in other disciplines such as classical music. So far, only little is known about the individual differences between musicians devoted to different music genres. Therefore, this study compared jazz musicians with musicians of classical and folk music with respect to their musical activities, creativity and personality.

Only few studies have investigated specific differences in attitudes, and learning approaches of musicians specialized in different music genres (e.g., Bézenak & Swindells, 2009; Creech et al., 2008; Papageorgi, Creech, & Welch, 2013; Welch et al., 2008). Classical musicians are reported to acquire musical skills mainly in formal educational settings involving one-to-one instruction and by practicing alone, whereas non-classical musicians devote more time to extra-curricular activities such as playing music for fun with others or having professional conversations (Bézenak & Swindells, 2009; Welch et al., 2008). Additionally, classical musicians attach greater importance on technical proficiency involving sight-reading, notation, and quality of tone, whilst non-classical musicians appear to attach greater importance to skills such as memorization or improvisation (Bézenak & Swindells, 2009; Creech et al., 2008). Bézenak and Swindells (2009) found that jazz musicians show higher intrinsic motivation and experience more pleasure in musical activities than classical musicians. In contrast, classical musicians report higher levels of performance anxiety than other non-classical musicians (Papageorgi et al., 2013). These findings already suggest important differences in the general approach towards learning and playing music between different genres such as jazz and classical music.

Research also addressed the question what factors lead to expert performance in music and more specifically in improvisational skills. It is now widely accepted that the cumulative amount of deliberate practice but also the quality of practice is highly predictive of mastery in the domain of music (Ericsson, Krampe, & Tesch-Römer, 1993; Williamon & Valentine, 2000). Additionally, there is evidence that individual differences in domain-general cognitive abilities also contribute to expert performance (Hambrick et al., in press). Beaty, Smeekens, Silvia, and Kane (in press) report a study where ten jazz students were video-taped during improvisation performances on a piece unknown to them, which then was rated for creativity by three professors of jazz studies. They found that creativity of improvisation was independently predicted by practice hours and divergent thinking ability (i.e., a common indicator of creative potential) of the jazz students. The findings suggests that divergent thinking, commonly defined as the ability to fluently generate original and appropriate ideas, may represent a relevant ability supporting improvisational creativity. This notion is in line with formal models of jazz improvisation stating that improvisation requires the continuous generation and evaluation of musical ideas (Pressing, 1988). Similarly, divergent thinking is considered as a central factor underlying creative thinking in music according to Webster’s model (2002), together with certain differences in personality and motivation. As a consequence, jazz musicians who are highly skilled in improvisation may differ in their creativity and personality from musicians of other genres. The aim of this study is to formally test this hypothesis by comparing Jazz musicians with musicians specialized in classical and folk music.

## 2 Materials and methods

### 2.1 Participants

A total of 120 students enrolled in the study of instrumental pedagogy at the University of Music and Arts in Graz participated in this study. They majored in various different musical instruments (e.g., piano, violin, voice), but were enrolled in one of three tracks related to a specific genre of music: classical music, jazz music, or folk music. The study curriculum is largely the same for all three genres, but classical and folk musicians have more courses on analyzing theoretical aspects of music as compared to Jazz musicians, who attend more courses focused on improvisational skills, ensemble playing and developing practical musical skills. The curriculum of folk musicians specifically requires the playing of at least two folk instruments and offers supplementary classes on folk dance or yodeling. We excluded seven participants who were enrolled in more than one music program and hence could not be attributed unambiguously to one music genre. Moreover, we included only students who indicated to have good to excellent language skills, leading to the exclusion of another 14 participants. The remaining sample consisted of 99 students, including 52 students of classical music, 25 students of jazz music, 21 students of folk music. On average, students had an age of 24.8 years (*SD* = 5.6), and have been studying music for 2.6 years (*SD* = 1.8). The sex distribution was fairly balanced with 47% females. The music groups did not differ in their age (*F*[2,95] = 2.25, *p* = .11), nor sex ratio (*χ*^2^[2] = .08, *p* = .96), but jazz students on average reported a longer duration of study (*F*[2,84] = 9.69, *p* = .001, partial-*η*^2^ = .19; classical music: 2.1 years; jazz music: 3.9 years; folk music: 2.4 years). For analyses involving speeded creativity tests we only included participants with German as mother tongue, resulting in 70 students (30 classical music, 22 jazz music, 18 folk music).

### 2.2 Psychometric tests and questionnaires

#### 2.2.1 Study and practice activities

We assessed relevant socio-demographic information including age, sex, nationality, selected study programs, and students gave a self-assessment of language skills (“excellent”, “good”, “fair”, or “bad”). They were asked how many hours they typically practiced their instruments at every single day of the week. This data was used to compute a reliable estimate of the practice hours per week. Finally, participants indicated how many concerts they play per semester, and how many competitions they had participated, how often they had won competitions, and how many productions they had published so far.

#### 2.2.2 Creativity assessment

Creative cognitive potential in the verbal domain was assessed with four divergent thinking tasks taken from a well-known German creativity test (Verbaler-Kreativitätstest; VKT; Schoppe, 1975). The tasks included two *alternate uses* tasks asking participants to generate different creative uses for a “tin can” and a “simple string”, and two *instances* tasks which asked to generate many things that could be used “for faster locomotion” or that are “bendable”. In all tasks, participants were instructed to find as many and as creative ideas as possible within the given time (120s, or 90s for the alternate uses and the instances task, respectively). The performance in the divergent thinking tasks was scored for ideational fluency (i.e., number of ideas), and ideational creativity. For the scoring of ideational creativity we created lists of pooled, alphabetically sorted, non-redundant responses for each task. Four experienced raters rated each idea for creativity on a four-point scale (“0, uncreative”, “1, somewhat creative”, “2, fairly creative”, and “3, very creative”). We then computed a top-3 creativity score by averaging the creativity ratings of the three top-most creative ideas within each task (Benedek, Mühlmann, Jauk, & Neubauer, 2013). This scoring method was found to yield valid scores that show to discriminant validity with regard to fluency measures (Benedek, Franz, Heene, & Neubauer, 2012; Benedek et al., 2013; Silvia et al., 2008). We averaged scores of the two alternate uses tasks and the two instances tasks to obtain one fluency score and one creativity score per task type. Additionally, creative potential in the figural domain was assessed with a picture completion task taken from the imagination subscales of the Berliner-Intelligenz-Test (Jäger, Süß, & Beauducel, 1997). Participants were shown a series of abstract lines which had to be completed in an original way to form meaningful objects. This task was scored for ideational fluency following the instructions of the test manual.

Besides creative potential, we also assessed real-life creative activities and achievements of the students using the inventory of creative activities and achievements (ICAA; described in Jauk, Benedek, & Neubauer, 2014). This inventory assesses creative activities and achievements in eight domains, including literature, music, arts and crafts, creative cooking, sports, visual arts, performing arts, and science and engineering. In the activities scale, participants report on a 5-point scale how often they carried out certain activities within the last 10 years. In the achievements scale, participants marked achievements they had already attained in each domain ranging from “I have never been engaged in this domain” (0 points) to “I have already sold some of my original work in this domain” (10 points), and values of all achievements are summed. Activities and achievements scores can be analyzed separately for each domain or as a composite score, after summing across domains.

#### 2.2.3 Personality assessment

Personality was assessed with respect to the Big Five using the NEO-FFI (Borkenau & Ostendorf, 1993). We assessed schizotypy using the German 17-item version of the Schizotypical Personality Questionnaire (SPQ; Klein, Andresen, & Jahn, 1997). Participants also completed the Error Orientation Questionnaire (EOQ; Rybowiak, Garst, Frese, & Batinic, 1999). This questionnaire contains 37 items asking about individual attitudes towards errors at work. In this study work was defined as practicing and performing activities as a musician. The EOQ consists of eight scales, including error competence, learning from errors, error risk taking, error strain, error anticipation, covering up errors, error communication and thinking about errors. Further questionnaires include the German version of the Frost Multidimensional Perfectionism Scale (FMPS; Stöber, 1998), the German Achievement Motivation Scale (AMS; Lang & Fries, 2006), rumination scale of the Perfectionism Inventory (PI; Hill et al., 2004), the student version of the SELLMO (a German questionnaire on learning and achievement motivation; Spinath, Stiensmeier-Pelster, Schöne, & Dickhäuser, 2012), and a set of self-devised questions on error behavior.

### 2.3 Procedure

Participants were tested in groups of 10–25 people in lecture rooms. First, they provided general information on their person, studies and practicing habits. They then worked on the divergent thinking tasks, the NEO-FFI, the SPQ, the EOQ, completed a self-devised questionnaire on error behavior, the ICAA, the FMPS, the AMS, the PI rumination scale and the SELLMO. The total session took about 90 min.

## 3 Results

Potential group differences between musical genres (classical music, jazz, or folk music) were analyzed by means of ANOVAs with the between-subject factor music genre. In case of significant group effects, LSD posttests were employed to further examine differences between group means.

### 3.1 Genre-related differences in general musical activities

Students of folk music devote on average 12 h per week on practice, which is significantly less than the typical practice periods of students of classical music (*p* = .01) or jazz music (*p* = .08), who spend about 18 h a week (*F*[2,92] = 3.39, *p* = .04, partial-*η*^2^ = .07; see Table 1). Jazz musicians played significantly more concerts per semester than classical musicians (*p* = .001) and folk musicians (*p* = .001; *F*[2,92] = 10.78, *p* = .001, partial-*η*^2^ = .19). On the other hand, jazz musicians participated in a lower number of music competitions (*F*[2,92] = 4.16, *p* = .02, partial-*η*^2^ = .08) than classical (*p* = .01) and folk musicians (*p* = .02), and also won a lower number of competitions (*F*[2,92] = 4.60, *p* = .01, partial-*η*^2^ = .09). Finally, folk musicians published a significantly higher number of works than classical musicians (*p* = .03; *F*[2,92] = 3.08, *p* = .05, partial-*η*^2^ = .06).

### 3.2 Genre-related differences in creativity

Students of classical, jazz and folk music were compared in their levels of creative potential, creative musical activities and creative musical achievements. As can be seen in Table 2, the three groups differed significantly in ideational creativity as measured by the alternate uses task (*F*[2,67] = 3.61, *p* = .03, partial-*η*^2^ = .10) and the instances task (*F*[2,67] = 3.96, *p* = .02, partial-*η*^2^ = .11). In both tasks, Jazz musicians showed higher ideational creativity than folk musicians (*p* = .01, and *p* = .01) and classical musicians (*p* = .09, and *p* = .02). No additional group differences were observed with respect to ideational fluency in the divergent thinking tasks (alternate uses task: *F*[2,67] = 0.34, *p* = .71; instances task: *F*[2,67] = 1.97, *p* = .15; picture completion task: *F*[2,67] = 0.17, *p* = .85).

We then analyzed potential group differences in creative activities and achievements with a focus on the musical domain. Jazz musicians reported to have engaged in a significantly higher number of creative musical activities over the last years (*F*[2,94] = 20.20, *p* < .001, partial-*η*^2^ = .30) than classical musicians (*p* < .001) and folk musicians (*p* < .001). Moreover, jazz musicians also showed higher creative achievements in the musical domain (*F*[2,94] = 15.03, *p* < .001, partial-*η*^2^ = .24) than classical musicians (*p* < .001) and folk musicians (*p* = .003). Notably, these group differences remained highly significant even after statistically controlling for differences in age and duration of study. As a side analysis, we also looked for group differences in other domains measured by the ICAA. The only significant finding was that folk musicians showed higher creative achievements in the domain of arts and crafts (*F*[2,94] = 8.92, *p* < .001, partial-*η*^2^ = .16) than classical musicians (*p* = .02) and jazz musicians (*p* = .02).

### 3.3 Genre-related differences in personality

Analyses of group differences in personality structure revealed a significant effect for extraversion (*F*[2,96] = 3.88, *p* = .02, partial-*η*^2^ = .08) and an effect by trend for openness (*F*[2,96] = 2.42, *p* = .06, partial-*η*^2^ = .05), but not effects for neuroticism, agreeableness, or conscientiousness (see Table 3). Specifically, folk musicians were found to be more extraverted than classical musicians (*p* = .007) and jazz musicians (*p* = .05). Classical musicians tend to be less open to new experiences than jazz musicians (*p* = .06) and folk musicians (*p* = .11). The music genre groups did, however, not differ in schizotypy (*F*[2,96] = 0.34, *p* = .72). No further significant group differences were observed in the motivational measures of this study, including all sub-facets of error orientation and perfectionism, and indicators of achievement motivation, learning motivation and rumination.

## 4 Discussion

The analysis of general musical activities revealed that the participants, although still studying at the Arts College, already were very accomplished musicians, completing a high weekly pensum of practice, playing a considerable number of concerts per year, regularly participating in music competitions, and having a substantial number of music productions published. Moreover, the music students already have attained very high levels of creative achievement in the domain of music, with 8% having accomplished every single musical achievement listed in the employed inventory. Interestingly, students of different music genres differed substantially in the relative amount of engagement in these activities. Classical musicians practice a lot and participate in a high number of competitions, but do not publish as many music productions as non-classical musicians. In contrast, jazz musicians perform a larger number of concerts per year but do not participate as often in music competitions. This confirms recent research showing that classical musicians are more focused on achievements related to solo professional work, whereas jazz musicians are more engaged in informal ways of practice by playing lots of concerts (Bézenak & Swindells, 2009; Creech et al., 2008; Welch et al., 2008). Finally, folk musicians showed a lower amount of weekly practice but have many performances in concerts, competitions and music productions. Taken together, these findings support the view that music learning is not only the product of formal educations systems but also includes different informal ways of practice (Green, 2002), and that approaches to music learning may differ between music genres (e.g., Welch et al., 2008).

Besides differences in musical activities, music genre was also related to individual differences in psychometric creativity. As expected, Jazz musicians showed higher divergent thinking ability (i.e., creative cognitive potential) in terms of ideational creativity than classical and folk musicians. The ability to fluently generate original ideas can be considered highly compatible with the improvisational skills that are required and trained in jazz music, and they may be of relatively lower significance in classical music or folk music (Pressing, 1988; Webster, 2002). Interestingly, Fink and Woschnjak (2011) reported a similar finding from the domain of dance. They found that modern/contemporary dancers, who are often required to improvise on stage, showed higher creative potential than ballet dancers who are normally obliged to adhere to well-structured choreographies. Of course, one can only speculate about the causality in the relationship between divergent thinking and improvisation abilities: Is high divergent thinking ability a precondition for becoming a good jazz musician, or does continuous improvisation training implicitly increase divergent thinking ability? There is evidence in support of both perspectives. On the one hand, divergent thinking was shown to predict improvisational creativity beyond the mere amount of practice (Beaty et al., in press). On the other hand, extensive engagement in divergent thinking can increase divergent thinking performance (Benedek, Fink, & Neubauer, 2006) and even have effects on relevant brain activation patterns (Fink, Grabner, Benedek, & Neubauer, 2006). Thus, both perspectives may apply to some extent. It would, however, need longitudinal studies to properly disentangle the relative effects in the causal relationship of creative potential and improvisation training.

As another result, jazz musicians reported to engage in a much larger number of creative activities in the domain of music than classical or folk musicians. In this context, it is important to point out that the employed measure of creative musical activities specifically reflects activities that involve creating something new but it does not consider playing music as a creative activity per se. Sample items of this scale include “I reinterpreted a piece of music in a creative way”, “I made up a melody”, “I made up a rhythm”, or “I artificially created sounds”. These creative activities are very common during jazz improvisation, whereas classical music usually involves a flawless reproduction with focus on technical excellence (Bézenak & Swindells, 2009; Creech et al., 2008). To be sure, playing e.g. a Bach sonata also involves individual expression by giving it a personal note in terms of temper and atmosphere, but this individuality usually does not go as far as changing the rhythm or melody of the piece in a substantial way. A similar argument may also explain why jazz musicians showed much higher creative achievements in the domain of music. Again, the scale explicitly focuses on achievements related to original pieces of work (e.g., musical compositions or rearrangements).

We also observed differences in personality between musicians of different genres of music. First of all, folk musicians are more extraverted than classical and jazz musicians. This finding may be related to the fact that folk music is commonly played at sociable events involving regular interactions with the audience. Therefore, the genre of folk music may more likely attract extraverted musicians that enjoy social interactions as an integral part of their performance. As an interesting additional finding, folk musicians were more achieved in the domain of arts and crafts. This may refer to stronger bonds of folk musicians to traditions and related skills in arts and crafts. We also observed a weak group effect for openness suggesting that jazz and folk musicians are more open to new experiences than classical musicians. Openness to new experiences reflects a preference for variety and the readiness to leave beaten paths. It is consistently related to creativity in the literature (e.g., Feist, 1998; Jauk et al., 2014) and may also promote the readiness to seek variation in musical play as required during improvisation.

Finally, we did not observe group differences in error orientation or motivational variables between music genres in this study. This is an interesting finding as one might have expected that jazz musicians e.g. are more comfortable with risk taking during their improvisational play. It should be noted, however, that the EOQ does not differentiate between errors occurring during practice or performance (Kruse-Weber & Parncutt, 2013). It hence is possible that the questions were rather attributed to the process of learning rather than to stage performances and thus errors were conceived as equally important.

## 5 Conclusions

This study revealed evidence that jazz musicians show higher divergent thinking ability, and a higher number of creative activities and achievements in the musical domain as compared to musicians from other genres such as classical music or folk music. These findings support the view that the music genre of jazz is highly associated with creativity, both in terms of musical activities and psychometric aspects of musicians. The observed differences may be related to differences in the formal and informal ways of practice and learning, with Jazz musicians attaching more importance on informal practice and playing for fun and lower value on technical perfection and competitions. Finally, the findings add to the evidence that individual differences in domain-general abilities (i.e., creative potential) may be relevant for the realization of domain-specific creative activities and achievements (Jauk et al., 2014; Kaufman & Beghetto, 2009).

## Figures and Tables

**Table 1:** Practice and performance activities in classical, jazz, and folk musicians.

	Classical music *M* (*SD*)	Jazz music *M* (*SD*)	Folk music *M* (*SD*)	Sign.
Amount of practice (hours per week)	18.7 (9.7)	17.4 (9.5)	12.3 (8.5)	⁎
Concerts played (per semester)	7.9 (8.4)	18.8 (13.8)	8.2 (7.3)	⁎⁎⁎
Music competitions participated	3.5 (3.7)	1.3 (1.7)	3.5 (3.3)	⁎
Music competitions won	1.5 (2.1)	0.3 (0.7)	0.8 (1.0)	⁎⁎
Music productions	1.6 (3.4)	3.4 (3.1)	4.3 (4.7)	+

*Note:* Sign. = Statistical significance.

⁎⁎⁎ *p* < .001.

⁎⁎ *p* < .01.

⁎ *p* < .05.

+ *p* < .10.

**Table 2:** Divergent thinking, creative musical activities and creative musical achievements in classical, jazz, and folk musicians.

	Classical music *M* (*SD*)	Jazz music *M* (*SD*)	Folk music *M* (*SD*)	Sign.
Alternate uses – Fluency	6.88 (2.49)	7.41 (2.18)	7.36 (2.97)	ns.
Alternate uses – Creativity	1.53 (0.34)	1.63 (0.24)	1.36 (0.36)	⁎
Instances – Fluency	10.18 (2.40)	11.39 (2.62)	9.89 (2.95)	ns.
Instances – Creativity	1.36 (0.26)	1.57 (0.30)	1.34 (0.32)	⁎
Picture completion – Fluency	6.53 (2.80)	6.18 (1.59)	6.28 (1.87)	ns.
Creative musical activities	13.98 (5.42)	21.48 (2.82)	14.10 (6.11)	⁎⁎⁎
Creative musical achievements	15.22 (12.78)	34.13 (15.86)	21.43 (16.21)	⁎⁎⁎

*Note:* Sign. = Statistical significance.

⁎⁎⁎ *p* < .001.

⁎ *p* < .05.

**Table 3:** Personality differences in classical, jazz, and folk musicians.

	Classical music *M* (*SD*)	Jazz music *M* (*SD*)	Folk music *M* (*SD*)	Sign.
Neuroticism	1.76 (0.67)	1.85 (0.69)	1.62 (0.86)	ns.
Extraversion	2.38 (0.48)	2.44 (0.42)	2.70 (0.37)	⁎
Openness	2.63 (0.51)	2.88 (0.50)	2.85 (0.53)	+
Agreeableness	2.71 (0.46)	2.86 (0.44)	2.73 (0.43)	ns.
Conscientiousness	2.80 (0.62)	2.85 (0.52)	2.81 (0.57)	ns.
Schizotypy	9.51 (3.00)	8.92 (3.48)	9.57 (3.20)	ns.

*Note:* Sign. = Statistical significance.

⁎ *p* < .05.

+ *p* < .10.
